# Assessing the causal link between liver function and acute pancreatitis: A Mendelian randomisation study

**DOI:** 10.1371/journal.pone.0300890

**Published:** 2024-04-05

**Authors:** Chun Zhang, Feng Lin, Deng-fang Guo, Qing-lin Wang, De-xian Xiao, Jian-yuan Lin, Shi Chen

**Affiliations:** 1 Department of General Surgery, Mindong Hospital Affiliated to Fujian Medical University, Ningde, Fujian, China; 2 Shengli Clinical Medical College of Fujian Medical University, Fuzhou, China; Shaanxi Provincial People’s Hospital, CHINA

## Abstract

A correlation has been reported to exist between exposure factors (e.g. liver function) and acute pancreatitis. However, the specific causal relationship remains unclear. This study aimed to infer the causal relationship between liver function and acute pancreatitis using the Mendelian randomisation method. We employed summary data from a genome-wide association study involving individuals of European ancestry from the UK Biobank and FinnGen. Single-nucleotide polymorphisms (SCNPs), closely associated with liver function, served as instrumental variables. We used five regression models for causality assessment: MR-Egger regression, the random-effect inverse variance weighting method (IVW), the weighted median method (WME), the weighted model, and the simple model. We assessed the heterogeneity of the SNPs using Cochran’s Q test. Multi-effect analysis was performed using the intercept term of the MR-Egger method and leave-one-out detection. Odds ratios (ORs) were used to evaluate the causal relationship between liver function and acute pancreatitis risk. A total of 641 SNPs were incorporated as instrumental variables. The MR-IVW method indicated a causal effect of gamma-glutamyltransferase (GGT) on acute pancreatitis (OR = 1.180, 95%CI [confidence interval]: 1.021–1.365, P = 0.025), suggesting that GGT may influence the incidence of acute pancreatitis. Conversely, the results for alkaline phosphatase (ALP) (OR = 0.997, 95%CI: 0.992–1.002, P = 0.197) and aspartate aminotransferase (AST) (OR = 0.939, 95%CI: 0.794–1.111, P = 0.464) did not show a causal effect on acute pancreatitis. Additionally, neither the intercept term nor the zero difference in the MR-Egger regression attained statistical significance (P = 0.257), and there were no observable gene effects. This study suggests that GGT levels are a potential risk factor for acute pancreatitis and may increase the associated risk. In contrast, ALP and AST levels did not affect the risk of acute pancreatitis.

## Introduction

Acute pancreatitis (AP) is a prevalent clinical condition, characterised by acute abdominal pain and elevated haematuric amylase levels, falling within the 3–5 range on the pancreatitis spectrum [1–3]. The worldwide incidence of AP is on the rise [4, 5]. Approximately 20–30% of AP cases progress to severe AP [6], with the potential to evolve into the life-threatening multiple organ dysfunction syndrome (MODS) [6, 7], associated with a mortality rate of 30–50% [2, 8]. In the context of AP, extrapancreatic organs (the liver in particular) exhibit early signs of damage, marked by varying increases in bilirubin, aminotransferase, and alkaline phosphatase (ALP) levels [9]. The liver performs critical physiological functions, including detoxification, metabolism, excretion, and biotransformation. Liver damage due to pathogenic factors leads to structural and functional alterations, resulting in clinical symptoms such as aminotransferase, coagulation mechanism disorder, and jaundice, which further aggravate the systemic inflammatory response of AP and adversely impact the overall prognosis of the disease. Hence, timely detection of liver function indicators is crucial for early assessment, clinical treatment, and prevention of AP.

Among various indicators of liver function, alanine transaminase (ALT) and aspartate aminotransferase (AST) are considered gold standards for assessing liver cell damage. ALP and gamma-glutamyltransferase (GGT) are classic indicators for cholestasis and are commonly used in the differential diagnosis of biliary and non-biliary pancreatitis. Notably, the acute onset of ALP is associated with AP [10]. Previous retrospective studies have shown significant elevations in serum levels of AST, ALT, ALP, GGT, and other liver function indices in AP cases [11]. Additionally, changes in these above indices tend to normalise after treatment. Furthermore, another cohort study found that various inflammatory factors were significantly elevated in patients with AP, except for liver function impairment [12]. Other scholars have dynamically observed changes in various biochemical indices in the early serum of AP rats, identifying several parameters, including AST, as potential predictive indicators for diagnosing AP [13]. In conclusion, changes in liver function are closely associated with the diagnosis and treatment of AP. However, these clinical observational studies are limited and susceptible to confounding factors. In the absence of high-quality randomised controlled trials, establishing a potential causal relationship between changes in liver function and AP remains challenging, necessitating further evidence.

Mendelian randomisation (MR) has been widely used in recent years for studying causality within genome-wide association study (GWAS) data. MR uses the characteristics of random division and combination with the formation of gametes with genetic variation to randomly group a population, theoretically avoiding the influence of confounding factors. The variation explained by genetic variation (the exposed instrumental variable) also takes precedence over the variation explained by the outcome, thereby eliminating the problem of reverse causality. Currently, MR is applied in the etiological screening of various complex diseases. Wan et al. [14] conducted a MR study on the causal relationship between leukocyte telomere length and prostate cancer, revealing a significant correlation. Specifically, each one-standard-deviation increase in genetically determined leukocyte telomere length was associated with an average 40.6% elevation in prostate cancer risk, yielding a mean odds ratio of 1.406 (95% CI [confidence interval]: 1.327 to 1.489, P< 0.001). Consequently, an extended genetically determined LTL was associated with a higher risk of PCa.

Notably, no MR studies pertaining to "liver function" and "AP" are available on the PubMed database. Therefore, this study capitalised on GWAS data, employing a genetic perspective to innovatively investigate causal relationships between different liver functions and AP through MR analysis.

## Materials and methods

### Data source

The data sources for ALP (ukb-d-30610_raw), AST (ukb—d—30650_irnt), GGT (ukb—d—30730_irnt), and AP (finn-b-K11_ACUTPANC) GWAS data were accessed through the UK Biobank (http://www.nealelab.is/uk-biobank) and FinnGen (https://www.finngen.fi/fi) websites. Notably, the deadline for accessing the data was 2023-07-15. The GWAS dataset relevant to this study was sourced from a public database, and informed consent was obtained from the study subjects in the original research. Therefore, ethical committee approval was not required for this particular aspect of the study.

### Single-nucleotide polymorphism as an instrumental variable condition setting and screening method

**Fig 1:**
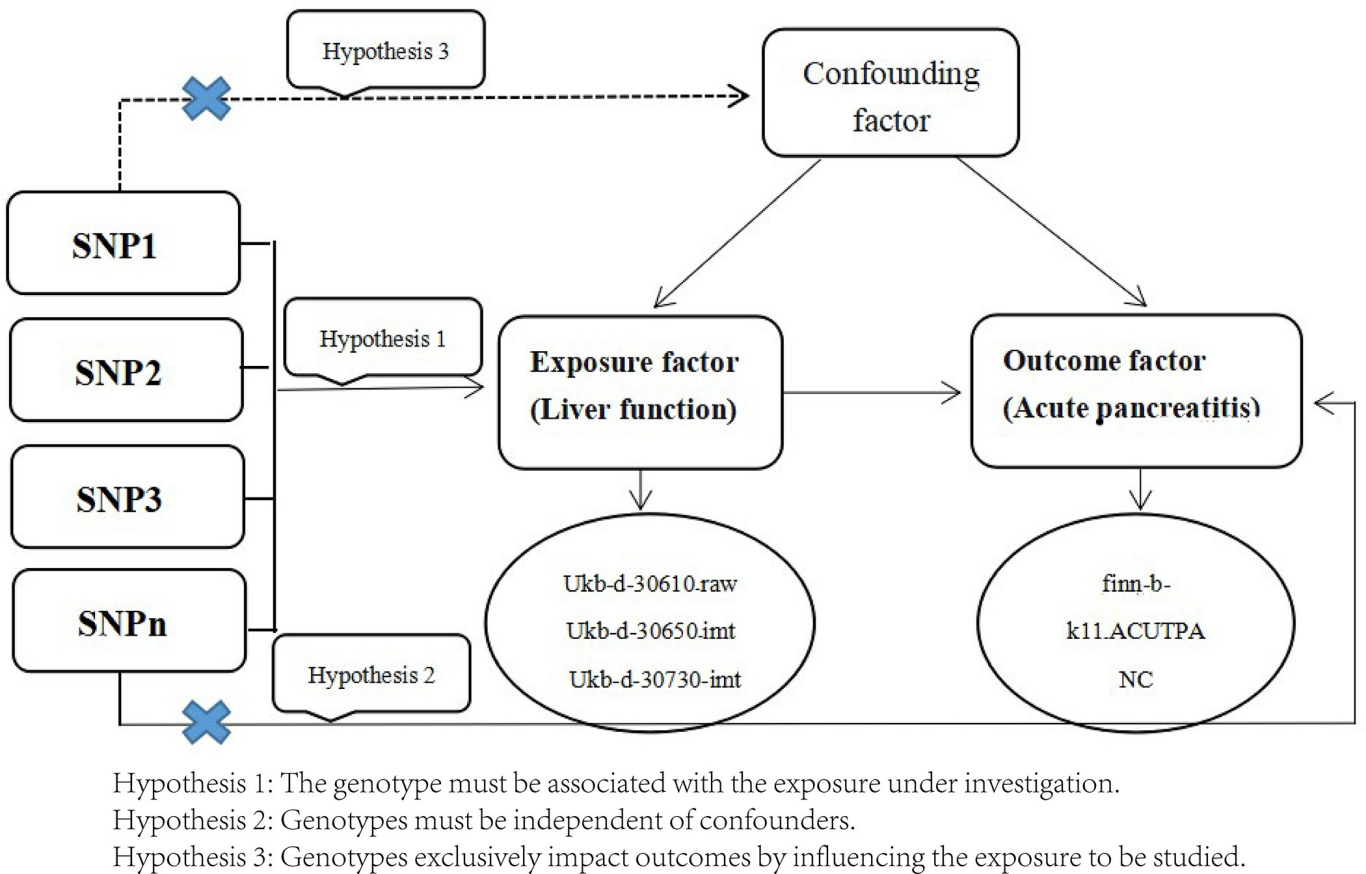
Mendelian randomisation schematic of the association between liver function and AP. Hypothesis 1: The genotype must be associated with the exposure under investigation. Hypothesis 2: Genotypes must be independent of confounders. Hypothesis 3: Genotypes exclusively impact outcomes by influencing the exposure to be studied. ukb-d-30610_raw, ukb-d-30650_irnt, ukb-d-30730_irnt, finn-b-K11_ACUTPANC. GWAS were the four published GWAS studies.

The three key assumptions of MR are strong correlation, exclusivity, and independence (Fig 1).

### Screening method

Meaningful single-nucleotide polymorphisms (SNPs) were screened from the aggregated GWAS data on liver function (P < 5× 10^−8^ as the screening condition; Hypothesis 1). The linkage disequilibrium coefficient (r^2^) was set to 0.001, and the linkage disequilibrium region width was set to 10000kb to ensure the independence of each SNP and exclude the influence of gene pleiotropy on the results. The SNPs associated with confounders and outcomes were eliminated using Pheno Scanner (Hypotheses 2 and 3). Relevant SNPs were extracted from the AP GWAS data. Setting the minimum r^2^ > 0.8, missing SNPs were replaced by SNPs with high linkage, and SNPs without substitution sites were deleted. Information from the two datasets mentioned above was summarised, and SNPs directly related to AP were excluded (P < 5× 10^−8^).

### Verification of causality

The results of the main analysis, heterogeneity analysis, and sensitivity analysis were included.

Five regression models were employed: the MR-Egger regression, inverse-variance weighted method (IVW), weighted median estimator (WME), weighted model, and a simple model. The SNP served as an instrumental variable for assessing the causal relationship between exposure (ALP, AST, and GGT) and the outcome (AP). Notably, the IVW method, which does not require individual-level data, directly calculated the causal effect value using summary data. The MR-Egger regression was used to calculate the correlation (Y) between each SNP and AP and to determine the correlation (X) between the SNP and ALP, AST, and GGT, which was then fitted into a linear function. The WME method estimated the causal effect of exposure on the outcome for the JTH SNP (β j). To assess the heterogeneity of the SNP, Cochran’s Q test was utilised. If heterogeneity existed, the results of the IVW model were considered. The intercept term of the MR-Egger method and leave-one-out detection were used for pleiotropic analysis. These methods were implemented using the TwoSampleMR package in R 4.2.2 software, with a significance level set at α = 0.05.

## Results

The data in this study were exclusively sourced from European individuals, both males and females, and the summary information is shown in Table 1. The data were drawn from four different publicly published GWAS databases, and informed consent was obtained from the subjects of the original study. As such, this part of the study did not require ethics committee approval.

**Table 1:** Brief information from the GWAS database for our MR study.

Variable	Sample size	SNP (per)	Crowd	Database	Sex	year
ALP	NA	13,586,006	Europe	UK Biobank	Male and female	2018
AST	NA	13,586,009	Europe	UK Biobank	Male and female	2018
GGT	NA	13,586,026	Europe	UK Biobank	Male and female	2018
Acute Pancreatitis	198166	16,380,428	Europe	FinnGen	Male and female	2021

Note: ALP, alkaline phosphatase; AST, aspartate aminotransferase; GGT, γ-glutamyltransferase; GWAS, genome-wide association study; SNP, single nucleotide polymorphism

After repeated screening, 641 SNPs were included into the AP dataset. Table 2 shows essential details from select SNPs. Notably, the F statistic corresponding to a single SNP ranges from 29.72 to 3345.31, indicating that a weak instrumental variable bias is less likely to affect the causal association.

**Table 2:** Basic information of some SNPs associated with AP.

SNP	CHR	POS	EA/OA	EAF	*β* ^a^	*SE*	*P*
rs28929474	14	94844947	T/C	0.020	3.027	0.221	1.17E-42
rs41282145	9	104249507	A/T	0.022	2.470	0.216	2.66E-30
rs55714927	17	7080316	T/C	0.192	2.361	0.079	4.94E-196
rs114114097	1	22072751	C/G	0.020	2.020	0.225	3.17E-19
rs72636979	11	296675	A/G	0.036	2.006	0.170	3.20E-32
rs11601507	11	5701074	A/C	0.068	1.749	0.122	8.78E-47
rs2207132	20	39142516	A/G	0.033	1.731	0.175	3.51E-23
rs114537356	1	22214279	A/C	0.021	1.688	0.219	1.23E-14
rs7949566	11	126285301	A/G	0.422	1.481	0.063	3.90E-122
rs149363012	12	670520	T/C	0.026	1.376	0.195	1.54E-12
. . .	. . .	. . .	. . .	. . .	. . .	. . .	. . .
rs182611493	19	19458388	G/A	0.012	-2.462	0.300	2.29E-16
rs41265999	1	22109952	A/G	0.020	-2.558	0.224	3.49E-30
rs4654748	1	21786068	T/C	0.474	-2.704	0.062	1.00E-200
rs111833688	3	12173015	C/T	0.007	-2.730	0.379	5.99E-13
rs112345326	1	22151854	A/G	0.017	-2.773	0.250	1.31E-28
rs9987289	8	9183358	G/A	0.908	-3.097	0.108	2.39E-181
rs145270291	1	20771550	A/G	0.004	-4.056	0.505	1.01E-15
rs56222534	1	21863905	C/T	0.069	-4.542	0.123	1.00E-200
rs550057	9	136146597	T/C	0.255	-6.549	0.071	1.00E-200
rs149344982	1	21889760	A/G	0.014	-15.532	0.269	1.00E-200

SNP, single-nucleotide polymorphism; CHR, chromosome; POS, population of interest; EA, effector allele; OA, other alleles; EAF, effector allele frequency; β, beta value; SE: β standard error; P, P value

^a^ SNPs are ranked in descending order by β value (allele effect value), P < 5×10^−8^.

### Causality verification

The regression results are shown in Table 3.

**Table 3:** MR regression causal association results of five methods.

Exposure factor	SNP number	Statistical method	*β*	*SE*	OR (95%CI)	*P*
ALP	209	MR-Egger regression	-0.006	0.003	0.994(0.988–1.001)	0.088
		WME analysis	-0.009	0.004	0.991(0.984–0.999)	0.019
		IVW method	-0.003	0.002	0.997(0.992–1.002)	0.197
		Simple model	-0.008	0.009	0.992(0.974–1.009)	0.358
		Weighted model	-0.006	0.003	0.994(0.988–1.001)	0.099
AST	202	MR-Egger regression	-0.117	0.160	0.889(0.650–1.216)	0.463
		WME analysis	0.081	0.151	1.084(0.806–1.458)	0.593
		IVW method	-0.063	0.086	0.939(0.794–1.111)	0.464
		Simple model	-0.069	0.343	0.933(0.476–1.829)	0.840
		Weighted model	0.110	0.191	1.116(0.767–1.623)	0.568
GGT	230	MR-Egger regression	0.080	0.135	1.083(0.831–1.412)	0.556
		WME analysis	0.175	0.119	1.191(0.944–1.503)	0.141
		IVW method	0.166	0.074	1.180(1.021–1.365)	0.025
		Simple model	0.338	0.236	1.403(0.883–2.228)	0.153
		Weighted model	0.161	0.125	1.174(0.919–1.500)	0.199

ALP, alkaline phosphatase; AST, aspartate transaminase; CI, confidence interval; GGT, gamma-glutamyl transferase; OR, odds ratio; SNP, single-nucleotide polymorphism; β, beta value; SE: β standard error; P, P value

**Fig 2:**
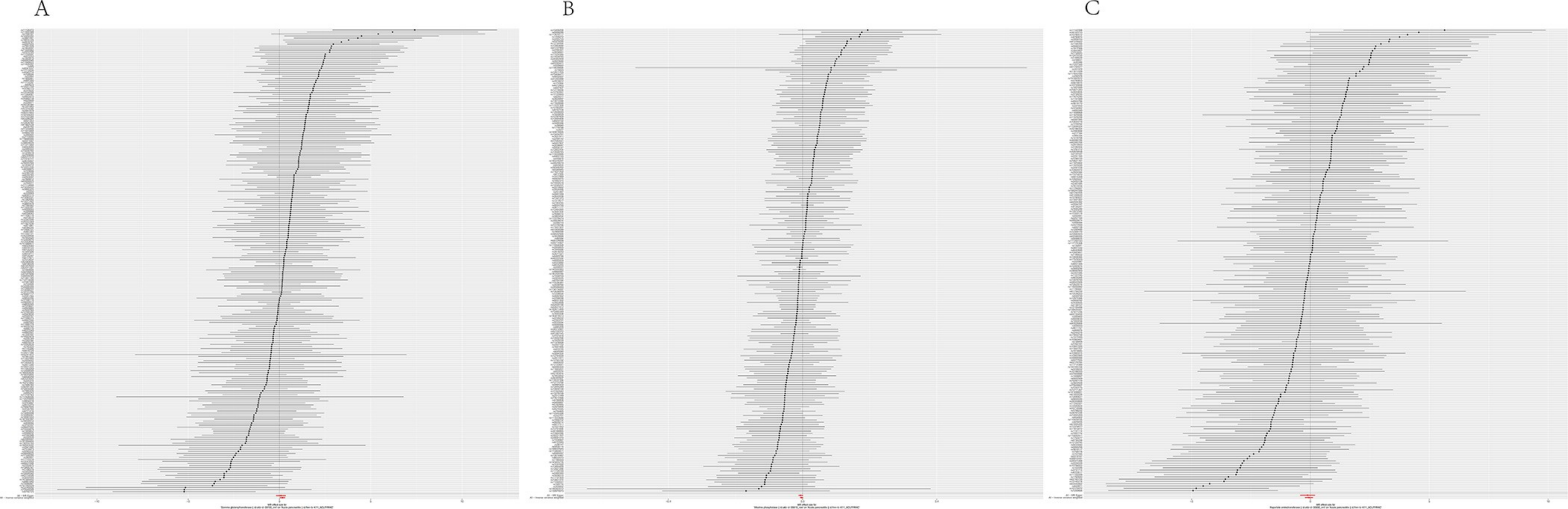
Forest map of the MR results. A: ALP, acute pancreatitis; B: AST, acute pancreatitis; C: GGT, acute pancreatitis. ALP, alkaline phosphatase; AST aspartate transaminase; GGT gamma-glutamyl transferase.

The results of the MR-Egger regression, the IVW method, the simple model, and the weighted model indicate no association between ALP and the incidence of AP. Specifically, the IVW model suggests that the risk of AP may decrease by 0.3% for each unit increase in ALP, but this difference lacks statistical significance (OR = 0.997, 95%CI: 0.992–1.002, P = 0.197> 0.05). Furthermore, the WME analysis suggests that ALP was a protective factor against the onset of AP (OR = 0.991, 95% CI: 0.984–0.999, P = 0.019). Combining the causal effect direction of the above methods, the IVW model results show no causal relationship between ALP and AP, indicating that ALP does not influence the incidence of AP. Similarly, findings from the MR-Egger regression, IVW method, WME analysis, simple model, and weighted model suggest no correlation between AST levels and the incidence of AP. Specifically, the IVW model indicates a potential decrease in the risk of AP by 0.63% with each unit increase in AST, although this difference remains statistically insignificant (OR = 0.939, 95% CI: 0.794–1.111, P = 0.464). Consequently, based on the causal effect direction determined by these methods, the IVW model results affirm the absence of a causal link between AST and AP, signifying that AST did not affect the incidence of AP. Furthermore, MR-Egger regression, WME analysis, simple model, and weighted model suggest that GGT is not associated with the incidence of AP. However, the IVW model suggested a statistically significant increase in the risk of AP by 1.18 times with each unit increase in GGT (OR = 1.180, 95% CI: 1.021–1.365, P = 0.025). Consequently, considering the causal effect direction elucidated by these methods, the IVW model results firmly establish a causal relationship between GGT and AP, implying that GGT will affect the onset of AP (Fig 2).

### Heterogeneity test

**Fig 3:**
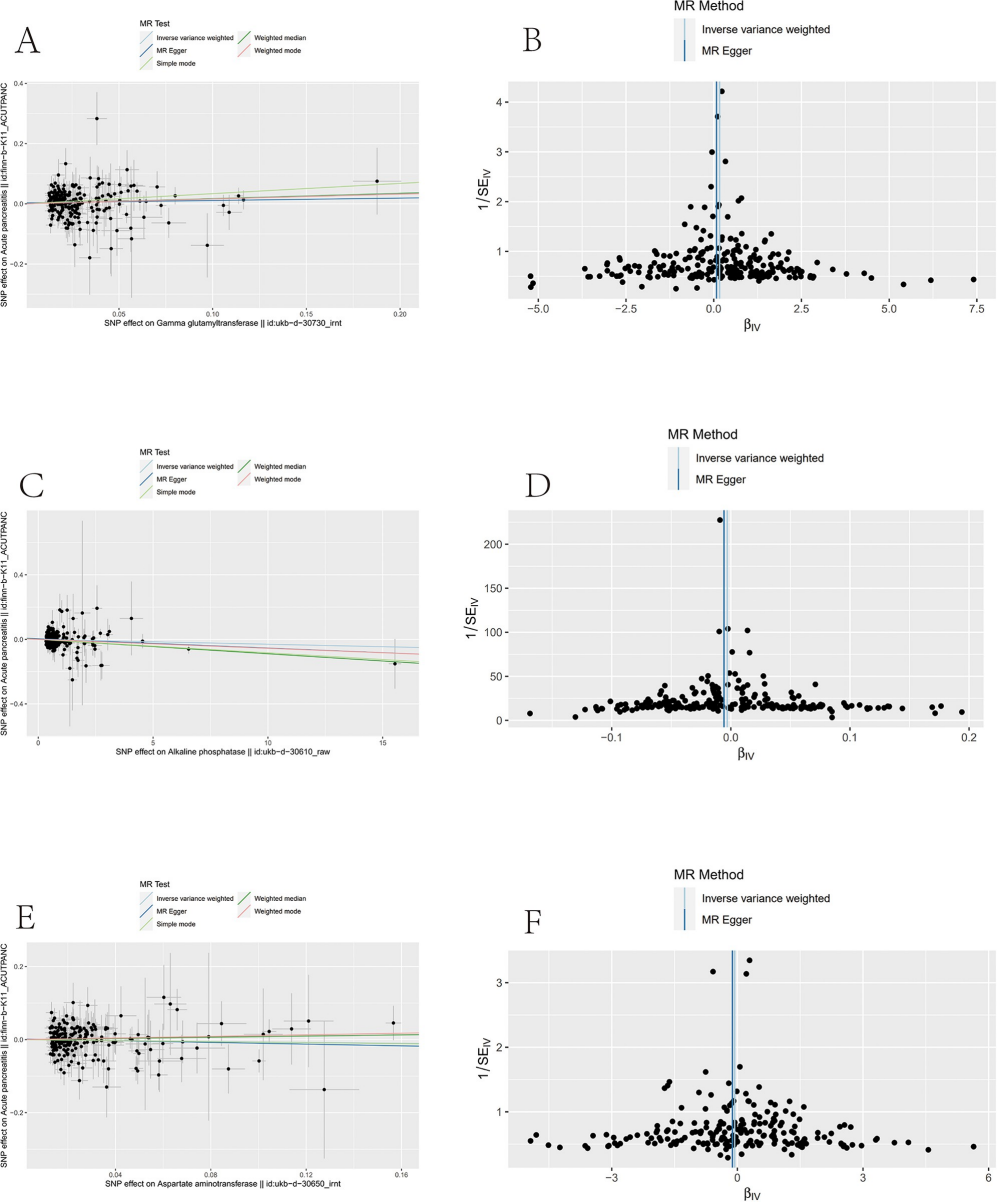
Mendelian randomisation scatter plot (left) and funnel plot (right). (A, B: ALP, acute pancreatitis) (C, D: AST, acute pancreatitis) (E, F: GGT, acute pancreatitis). ALP, alkaline phosphatase; AST aspartate transaminase; GGT gamma-glutamyl transferase.

The Cochran Q test for MR-Egger regression and the IVW method showed that the Q values for ALP, AST, and GGT-AP were all > 0.05, indicating an absence of heterogeneity among the SNPs (Table 4). However, statistical significance was not observed between the intercept term of the MR-Egger regression and zero (all P > 0.05). Consequently, we conclude that there was no evidence of gene pleiotropy in the SNPs (Table 5). Scatter and funnel plots for ALP, AST, and GGT showed a symmetrical distribution of all included SNPs, suggesting that causal associations were less likely to be affected by potential bias (Fig 3).

**Table 4:** Results of the MR heterogeneity analysis.

Exposure factor	Statistical method	Statistic Q	*P*
ALP	MR-Egger regression	220.310	0.250
	IVW method	221.687	0.245
AST	MR-Egger regression	209.145	0.314
	IVW method	209.318	0.329
GGT	MR-Egger regression	252.653	0.126
	IVW method	253.296	0.130

ALP, alkaline phosphatase; AST, aspartate transaminase; GGT, gamma-glutamyl transferase.

**Table 5:** MR-Egger regression analysis of instrumental variables.

Exposure factor	Intercept	*SE*	*P*
ALP	0.004	0.003	0.257
AST	0.002	0.004	0.685
GGT	0.003	0.004	0.447

ALP, alkaline phosphatase; AST aspartate transaminase; GGT gamma-glutamyl transferase; SE: standard error; P, P value

### Sensitivity analysis

**Fig 4:**
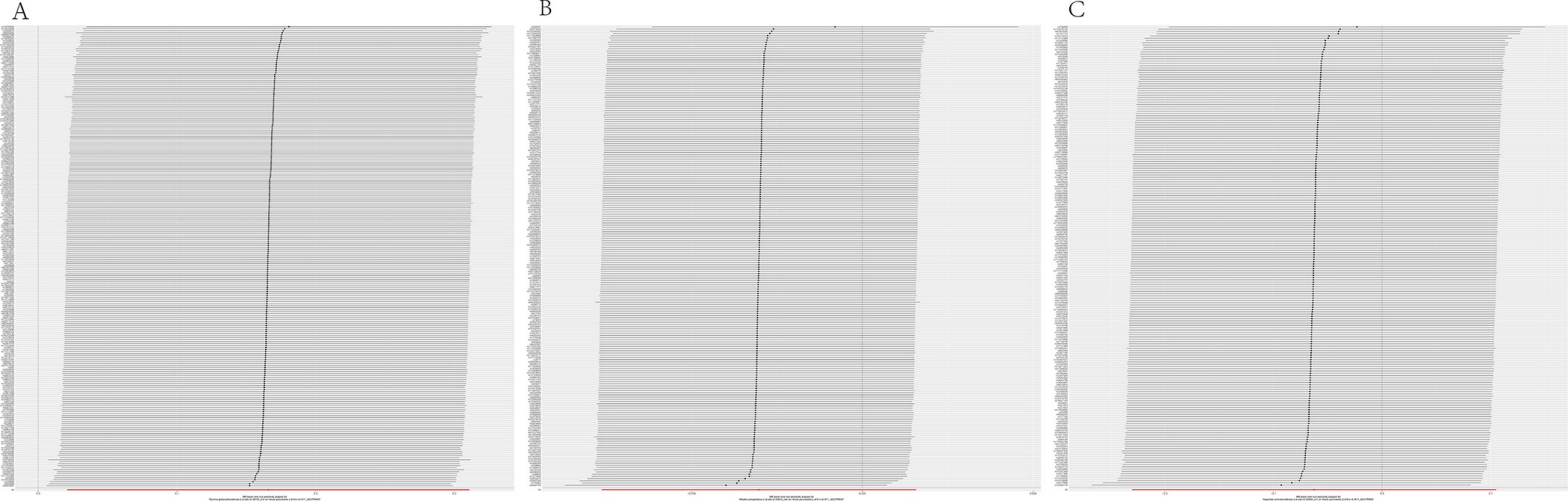
Results of the "Leave-one-out" sensitivity analysis. A: ALP, acute pancreatitis; B: AST, acute pancreatitis; C: GGT, acute pancreatitis). ALP, alkaline phosphatase; AST aspartate transaminase; GGT gamma-glutamyl transferase.

After conducting the leave-one-out test and sequentially removing ALP, AST, and GGT, the analytical results for the remaining SNPs were similar to those of all SNPs included (Fig 4). Notably, no SNPS exhibited a significant influence on the estimation of the causal association, indicating that the MR results of this study were robust.

## Discussion

This is the first study to investigate the causal relationship between liver function and AP using MR Analysis. Our findings revealed that GGT is a risk factor for AP, increasing the susceptibility to this condition by approximately 1.18 times. However, no causal relationship was noted for ALP and AST with AP, indicating that the levels of ALP and AST do not affect the incidence of AP.

Previous studies have shown that AP often induces alterations in serum biochemical liver indices, which can subsequently impact the severity and prognosis of the condition. Elevated levels of ALT and AST often serve as early indicators of liver disease severity and usually precede clinically abnormal symptoms. The levels of serum ALT and AST are positively correlated with the severity of pancreatitis and tend to return to normal after the pancreatitis is cured [15]. ALP primarily exists in the bile capillaries of the liver, bone, kidney, and placenta, whereas GGT mainly localises in the cell membranes of the liver, pancreas, spleen, kidney, heart, and brain. Any degree of hepatic parenchymal damage, cholestasis, or obstruction within the common bile duct, starting from the capillary bile duct, may result in elevated ALP and GGT levels. In gallstone-related AP, patients with higher levels of GGT, bilirubin, and ALP experienced prolonged hospitalisation and a higher risk of liver failure and mortality compared with those in individuals without these elevations [16, 17]. Most previous studies were either observational or retrospective, thus failing to shed light on the underlying mechanisms responsible for hepatic injury-associated AP. This study found evidence supporting a causal relationship between GGT levels and AP. Notably, GGT levels emerge as a risk factor for AP, aligning closely with findings from previous studies. Although GGT may contribute to biliary AP, there is no causal relationship between ALP, AST, and AP.

The main manifestations of liver damage are liver enzymological changes, abnormal bilirubin metabolism, dysfunction of substance synthesis, and decreased biodegradation. Abnormal liver function can lead to various complications, including AP. Hyperlipidaemia-induced AP can promote the release and activation of lipase, catecholamine, glucagon, and growth hormones, thereby accelerating the metabolism of fats within organs and tissues, leading to their release into the bloodstream. Consequently, this further increases the blood lipid concentrations, thus forming a vicious cycle between hyperlipidaemia and AP [18]. Studies have shown that oxidative stress plays an important role in the pathogenesis of AP. The potential effect of oxidative stress on the poor antioxidant status of the pancreas can lead to pancreatic cell damage. In a study conducted by Ściskalska et al. [19], it was demonstrated that cigarette smoke-induced oxidative stress played an important role in the progression of inflammation in patients with pancreatitis. One of the many enzymes involved in isogenic detoxification is GGT, which is essential for maintaining glutathione homeostasis [20]. Its primary physiological function is to make cysteine available for the regeneration of intracellular GSH, thereby protecting the cells from oxidative stress. GGT also participates in the transport of amino acids across cell membranes, leading to the formation of cysteine glycine, which undergoes a strong (Reduction-Oxidation) REDOX reaction and produces free radicals. Therefore, GGT participates in the production of free radicals, which can be considered markers of oxidative stress [21, 22]. These mechanisms may explain why GGT affects the occurrence of AP.

ALP, ALT, AST, and TBil are serological indicators currently used in clinical practice to reflect liver injury, and studies have shown that indicators of liver damage correlate with the severity of AP disease [23]. However, there is no evidence that the ALP and AST levels are risk factors for AP, which is consistent with the results of our Mendelian analysis. In AP, the balance between oxidant and antioxidant systems is destroyed, (Oxygen Free Radical)OFR mediates the increase in lipid peroxidation in pancreatic tissues [24], and lipid peroxidation is significantly enhanced not only in pancreatic tissues but also in liver tissues. Therefore, endotoxins and lipid peroxidation may be the mechanisms of liver injury induced by oxidative stress [25, 26]. The disturbance of pancreatic and hepatic microcirculation not only affects the blood supply to other organs but also leads to increased concentrations of inflammatory factors and active peptides within tissues and cells. This exacerbates the state of ischaemia and hypoxia in pancreatic and hepatic cells and tissues, further aggravating the functional damage of the pancreas and liver [27].

The advantages of this study are as follows. First, this study is the first to use MR methods to investigate the causal relationship between liver function and AP, thereby expanding the horizons beyond traditional observational studies. Second, unlike previous single-outcome MR studies, our research also examines the causal relationship between ALP, AST, GGT, and AP. This offers valuable directional references for subsequent studies. Third, the exposure and outcomes were obtained from European populations, thus avoiding population bias. Fourth, this was a study with a large sample size using a variety of MR research methods with high statistical efficacy and reliability. To our knowledge, this represents the first instance of MR being applied to investigate the causal relationship between liver function and AP. To support the robustness of our findings, we conducted a comprehensive set of MR estimates and sensitivity analyses, encompassing IVW, WM, MR-Egger, Cochran’s Q test, and leave-one-out analysis. These analyses collectively confirmed the reliability and stability of our results.

However, this study has some limitations. First, this study only verified the causal relationship between ALP, AST, GGT and AP. To comprehensively understand the causal relationship between AP and other liver function indicators, further investigations are required. Second, all GWAS data were exclusively sourced from European populations. Consequently, our results are not representative of the entire population, and the causal relationship between liver function and AP in other populations remains to be studied. Third, the GWAS database lacked detailed demographic characteristics and clinical data. The absence of individualised data prevented us from stratifying our analysis according to sex, age, and other pertinent factors.

This MR Study found that GGT is a inducing factor of acute pancreatitis. It has certain guiding significance for clinical practice, It is suggested that patients with high GGT should avoid exposure to the causes of acute pancreatitis (such as avoiding overeating, heavy drinking, etc.), and patients with acute abdominal pain accompanied by high GGT should be highly alert to acute pancreatitis, and be informed in advance and intervene early. The results of this study only provide preliminary evidence and guidance direction, and further clinical studies are needed to explore and verify the diagnostic weight of GGT elevation.

## Conclusions

Our MR study demonstrated that GGT is a significant risk factor for AP, while no causal relationship was observed for ALP and AST with the occurrence of AP. Furthermore, ALP and AST do not exert any influence on the development of AP. This study provides certain reference values for the prevention and treatment of clinical AP. Future studies are needed to further elucidate the mechanism by which liver function impacts AP and to validate these findings using data that are more consistent with clinical diagnoses.

## Supporting information

S1 Checklist STROBE-MR-checklist. (DOCX)

S2 Checklist PLoS one human subjects research checklist. (DOCX)

S1 Text Deleted SNPS of merged data. (TXT)

S1 Table F-statistic. (CSV)

S2 Table My data. (CSV)

S3 Table OR data. (CSV)

S4 Table Heterogeneity. (CSV)

S5 Table Pleiotropy. (CSV)

S6 Table Confounding factor. (XLSX)
